# Range contractions of the world's large carnivores

**DOI:** 10.1098/rsos.170052

**Published:** 2017-07-12

**Authors:** Christopher Wolf, William J. Ripple

**Affiliations:** Global Trophic Cascades Program, Department of Forest Ecosystems and Society, Oregon State University, Corvallis, OR 97331, USA

**Keywords:** carnivore guild, intact guild, predator, geographical range, livestock, historic

## Abstract

The majority of the world's terrestrial large carnivores have undergone substantial range contractions and many of these species are currently threatened with extinction. However, there has been little effort to fully quantify the extent of large carnivore range contractions, which hinders our ability to understand the roles and relative drivers of such trends. Here we present and analyse a newly constructed and comprehensive set of large carnivore range contraction maps. We reveal the extent to which ranges have contracted since historical times and identify regions and biomes where range contractions have been particularly large. In summary, large carnivores that have experienced the greatest range contractions include the red wolf (*Canis rufus*) (greater than 99%), Ethiopian wolf (*Canis simensis*) (99%), tiger (*Panthera tigris*) (95%) and lion (*Panthera leo*) (94%). In general, the greatest range contractions occurred in Southeastern Asia and Africa. Motivated by the ecological importance of intact large carnivore guilds, we also examined the spatial extent of intact large carnivore guilds both for the entire world and regionally. We found that intact carnivore guilds occupy just 34% of the world's land area. This compares to 96% in historic times. Spatial modelling of range contractions showed that contractions were significantly more likely in regions with high rural human population density, cattle density or cropland. Our results offer new insights into how best to prevent further range contractions for the world's largest carnivores, which will assist efforts to conserve these species and their important ecological effects.

## 1. Introduction

Large carnivores are among the world's most threatened species [1]. They face a wide variety of anthropogenic threats including persecution by humans, particularly over livestock-related conflicts, hunting and trapping, and loss of prey base [1,2]. Moreover, their unique life-history characteristics (e.g. relatively long gestation lengths among carnivores) make them particularly vulnerable to anthropogenic threats associated with increasing human population densities [3]. There is now extensive literature documenting the ecological importance of these species, with trophic cascades having been found for seven of the 31 large carnivores [1]. However, research into the ecological effects of large carnivores has almost certainly been hampered by the limited knowledge available on the extent to which these species have undergone range contractions.

Increases in species extinction risk are typically linked to the loss of individual populations and associated declines in geographical range [4]. Thus, species' range contractions are closely related to extinction risk and the analysis of range contractions can provide spatially explicit insight into what is happening to a species both at the level of individual populations and as a whole. They have major conservation value in terms of guiding efforts to limit further range contractions, and potentially, promoting range expansions within historic ranges. Analyses of range contractions often consider the spatial patterns of the contractions, with emphasis on the extent to which ranges contract towards their centre [5,6]. Such analyses can inform conservation decisions regarding the most critical regions of a species range to protect [7]. An alternative form of range contraction analysis involves modelling the likelihood of range contraction using spatially varying predictor variables like human footprint metrics [8]. Such models can help conservation researchers gain a better understanding of the roles and relative influence of potential drivers of range contractions.

There are several major multispecies range contraction results that include some of the extant terrestrial large carnivores. An extensive set (*n* = 173) of terrestrial mammals have together lost more than 50% of their historic range, with losses most severe in regions with high human population density (HPD) or other human impacts [4]. Among 43 ungulate and carnivore species in North America, 17 species have experienced range contractions of at least 20%, and range contractions have been most common in regions with high human influence [9]. Similarly, generally positive relationships have been found between HPD and the probability of range contraction for 10 large carnivores in portions of their ranges [10]. Among 245 species spanning numerous taxonomic classes, most species tended to persist in the peripheral regions of their ranges rather than the historic core [11]. However, among mammals, biome type has been found to be more predictive of the likelihood of range contraction than position within range (distance to historic centroid) [8]. Currently, less than 21% of the world's land retains its historic large (greater than 20 kg) mammal guild [12]. Moreover, large carnivore guilds have undergone a substantial loss in functional diversity since the Late Pleistocene [13].

While each of these range contraction analyses has included some of the large carnivores, no analysis has yet focused on range contractions of all extant terrestrial large carnivores worldwide. Here, we conduct the first such global analysis of large carnivore range contractions. We used historic and current range maps for the large carnivores (greater than or equal to 15 kg body mass) with reliable historic range maps available. We excluded the otters (*Lutrinae*) and polar bear (*Ursus maritimus*) as these species are primarily aquatic and our analysis focuses on terrestrial species. We excluded the maned wolf (*Chrysocyon brachyurus*) as an accurate historic range map was not available for this species. This was the only species that we excluded from our analysis due to lack of a suitable historic range map. Guided by the range contraction literature, we split our analysis into several research questions and hypotheses. We hypothesized that range contractions have been greatest (in terms of numbers of species lost) in sub-Saharan Africa, Southern Asia and Southeastern Asia because these regions have historically contained many large carnivores. Given the similarities among large carnivore species, we hypothesized that they have had major range contractions regardless of life-history traits. We further hypothesized that intact large carnivore guilds are very uncommon and occupy small fractions of their historic areas, with most intact guilds tending to contain few species and occurring at high latitudes where human influence is lower. Finally, we hypothesized that high HPD, cropland and cattle density are all positively correlated with the likelihood of range contraction as prior analyses suggest human influence in general is a key driver of range contractions [4,9].

## 2. Material and methods

### 2.1. Historic and current range maps

We obtained current range maps for 24 of the 25 large carnivores in our analysis from the International Union for Conservation of Nature (IUCN) Red List [14]. The current range map of the dingo (*Canis dingo*) was provided by Letnic *et al*. [15]. For the current ranges using IUCN source maps, we treated the ranges as areas where species are classified as ‘extant’ or ‘probably extant’ (regardless of origin).

For the historic range maps, we used maps from a variety of sources (electronic supplementary material, table S1). We treated the historic maps as corresponding to *ca* AD 1500 after Morrison *et al*. [12].

When comparing current and historic range maps, we frequently observed ‘slivers’ (long regions of apparent range expansion next to historic ranges) and ‘islands’ (isolated areas of apparent range expansion near historic ranges). As these slivers and islands are more likely artefacts associated with mapping errors than real range expansions, we extended the historic ranges to include all areas in the current range of each species. We made slight adjustments to the historic and current ranges near coastlines in order to align them with each other and a map of land, adding terrestrial regions within three 0.05° raster grid cells of each range and the ocean to each range. We then clipped ranges using species altitude limits from the Red List species fact sheets when these data were available. We did not do this for the Ethiopian wolf (*Canis simensis*) as its elevation limit appears to be for its current range only. Minor additional modifications were made to the historic range maps on a case by case basis (electronic supplementary material, table S1).

### 2.2. Mapping

We added the species ranges (0.05° resolution) together to form composite richness maps corresponding to historic species richness, current species richness, species richness lost (historic minus current) and percentage of species lost. We also quantified the change in large carnivore richness at the scales of biomes and geographical regions [16,17]. We assessed the extent of intact carnivore guilds by defining regions with intact guilds to be those with zero carnivores lost. Geographic Information System (GIS) analysis was done in ArcGIS 10.1 and ‘R’ [18,19].

### 2.3. Modelling range contractions

We modelled range contractions for all species together using a residuals auto-covariate (RAC) model [20,21]. We used composite species range maps at 50 km resolution because it was considered most appropriate given the accuracy of the historic range maps [8]. Observations in our model were binary, with each observation corresponding to whether or not a species’ range had contracted from a 50 × 50 km grid cell within its historic range.

We included a spatial auto-covariate term in order to account for potential autocorrelation. The spatial auto-covariate was derived from the corresponding non-spatial generalized linear mixed model deviance residuals. Specifically, it was based on the average of the residuals for all grid cells within 300 km of each grid cell and was calculated using the ‘spdep’ R package [22]. Additionally, we included random intercepts at the level of species to account for potential taxonomic dependence. We fit the models using the ‘glmer’ function in the ‘lme4’ R package [23].

For predictor variables, we used 2014 estimated cattle density (cattle per square kilometre) from the Food and Agriculture Organization's (FAO) gridded livestock of the world database [24,25], 2015 estimated rural HPD [26] and cropland [27] (electronic supplementary material, figure S1). All predictors were included in the model together and were standardized to have mean = 0 and s.d. = 1 so that estimated effect sizes were comparable. To explore the extent to which the estimated effects vary by region, we also fit a model including random intercepts and slopes by geographical region. We quantified the effect of these additional terms by calculating the change in conditional (i.e. accounting for the random effects) pseudo-*R*^2^ using ‘r.squaredGLMM’ in the ‘MuMIn’ R package [28] and by calculating the random effect estimates (conditional modes). To visualize these results, the random effect estimates were added to the fixed effect estimates, with 95% prediction intervals constructed under the assumption that random and fixed effect estimates are independent. Finally, we quantified the effect of variability at the species level by looking at the difference between marginal and conditional pseudo-*R*^2^ for our main model.

## 3. Results

**Figure 1.**
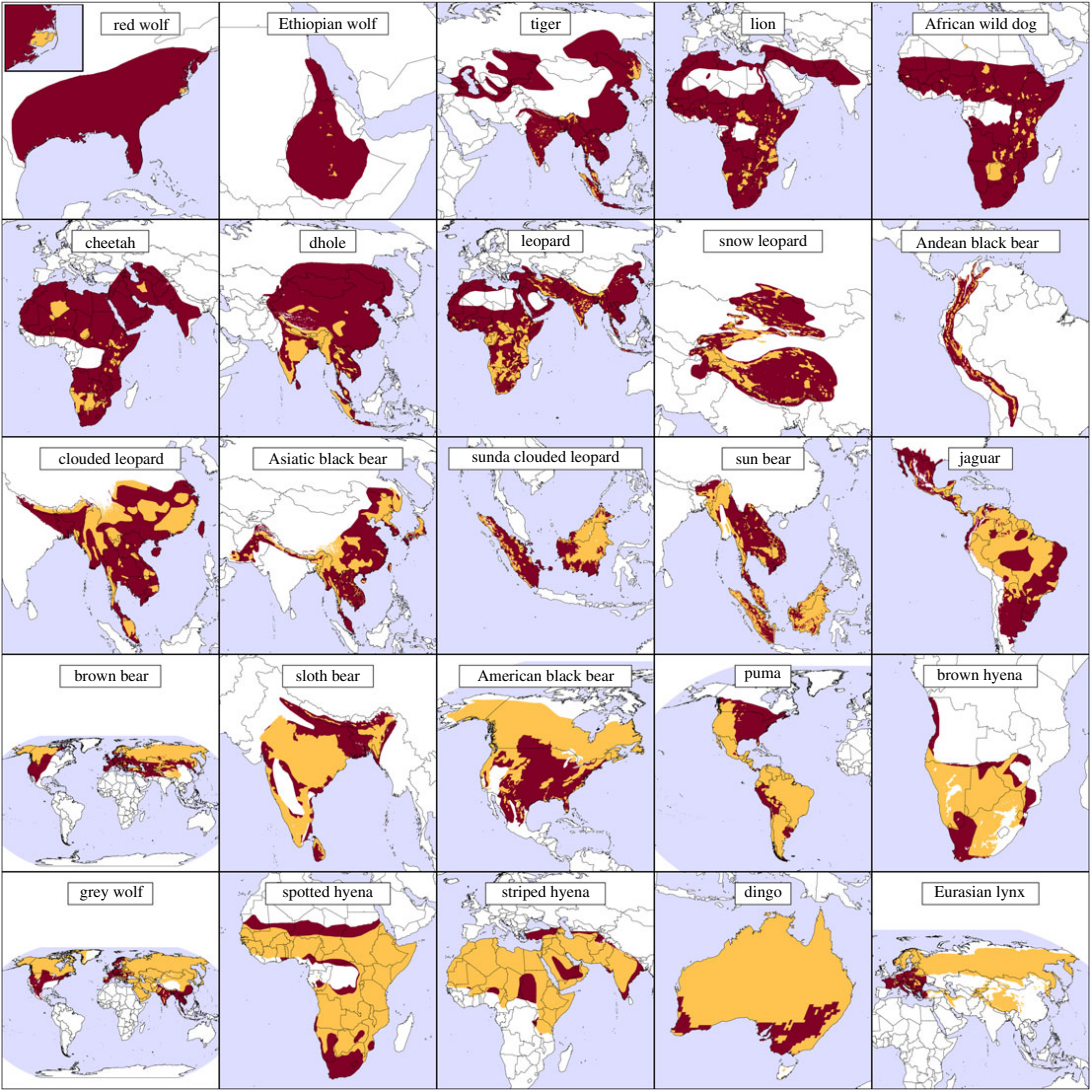
Range contraction maps for 25 large carnivores. Regions of persistence (i.e. inside both historic and current ranges) are shown in yellow-orange, while regions of contraction (inside historic but not current range) are shown in dark red. Species are ordered by percentage range contraction with the greatest contractions shown in the uppermost panels.

**Figure 2.**
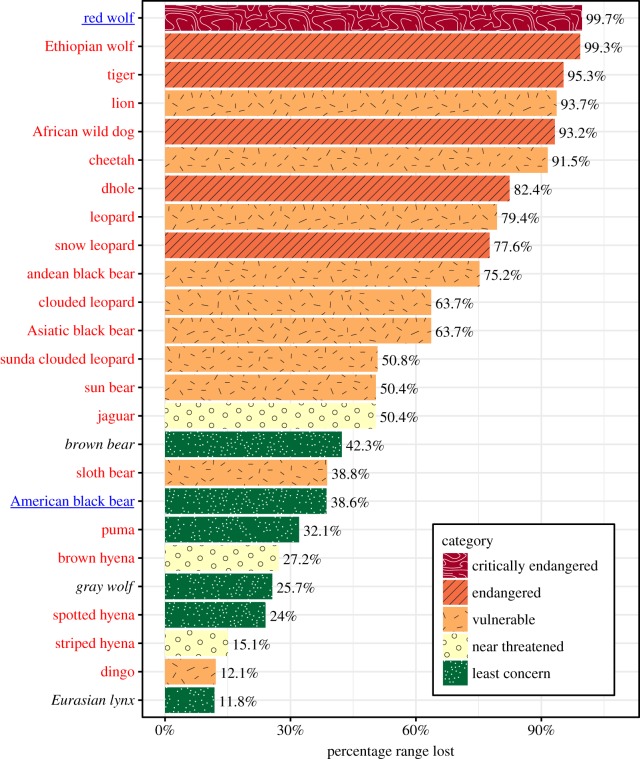
Percentage of historic range lost for each large carnivore. Carnivores names are coloured by population trend (red, decreasing; black italics, stable; blue underlined, increasing) and bar colours indicate carnivore endangerment status.

The compiled set of carnivore range contraction maps (*n* = 25) showed significant range contractions for many large carnivore species (figure 1; electronic supplementary material, table S2). The six large carnivores with the greatest estimated range contractions were the red wolf (greater than 99%), Ethiopian wolf (99%), tiger (*Panthera tigris*) (95%), lion (*Panthera leo*) (94%), African wild dog (*Lycaon pictus*) (93%) and cheetah (*Acinonyx jubatus*) (92%), while the six carnivores with the smallest range contractions were the Eurasian lynx (*Lynx lynx*) (12%), dingo (12%), striped hyena (*Hyaena hyaena*) (15%), spotted hyena (*Crocuta crocuta*) (24%), grey wolf (*Canis lupus*) (26%) and brown hyena (*Parahyaena brunnea*) (27%) (figure 2). With the exception of the red wolf, all 13 of the large carnivore species that experienced the greatest percentage range contraction are currently both threatened with extinction (IUCN Red List status ‘Vulnerable’, ‘Endangered’ or ‘Critically endangered’) and have decreasing population trends according to the IUCN Red List (figure 2). Overall, the extent of range contractions did not appear to vary substantially with large carnivore mass or taxonomic family although the hyenas experienced relatively minor range contractions as a group (electronic supplementary material, figure S2).

**Figure 3.**
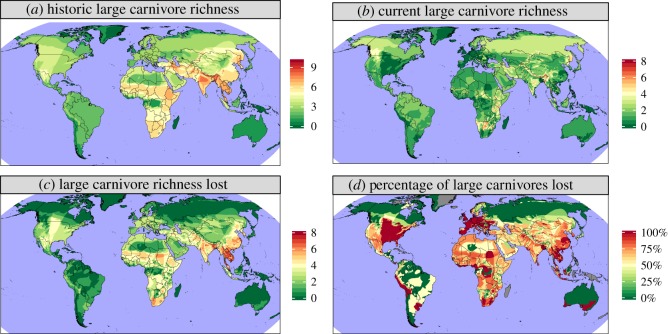
Composite range contractions maps based on all 25 large carnivores. Variables shown are (*a*) historic species richness, (*b*) current species richness, (*c*) their difference (i.e. lost species richness) and (*d*) the percentage of species lost.

**Figure 4.**
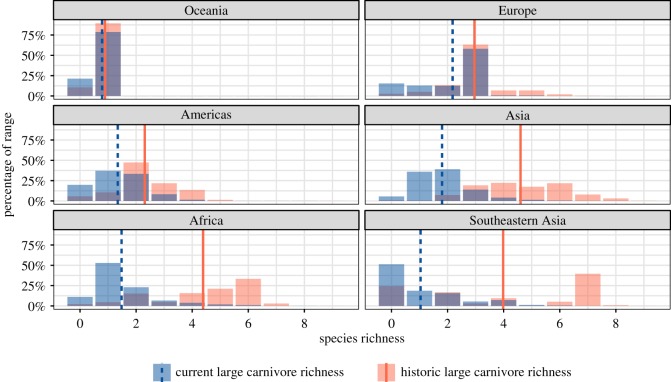
Current and historic species richness histograms by region of the world. ‘Asia’ excludes Southeastern Asia, which is shown separately. Vertical lines indicate mean richness. Panels are sorted by difference in mean richness and indicate that the most extensive range contractions (by this metric) occurred in Southeastern Asia, Africa and the rest of Asia. Overlap between current and historic range histogram bars is shown in dark purple.

The composite range contraction maps showed the highest historic large carnivore richness in South and Southeast Asia (up to nine co-occurring species) and Africa (up to six co-occurring species) (figure 3). The greatest declines in average large carnivore richness were observed in Southeast Asia (2.9 species), Africa (2.9) and Asia (excluding Southeast Asia) (2.8), while the smallest declines were observed in Oceania (0.1), Europe (0.8) and the Americas (1.0) (figure 4). By contrast, regions with high percentages of large carnivores lost were more uniformly distributed spatially, with particularly large areas where 100% of historic large carnivores have been extirpated occurring in Europe, the Eastern United States and Southeast Asia (figure 3). In terms of biomes, the greatest declines occurred in ‘Tropical & Subtropical Dry Broadleaf Forests' (3.0 species), ‘Flooded Grasslands and Savannahs' (2.6) and ‘Tropical and Subtropical Grasslands, Savannahs and Shrublands' (2.6), while the smallest declines occurred in ‘Tundra’ (0.1), ‘Boreal Forests/Taiga’ (0.4) and ‘Temperate Conifer Forests' (1.6) biome types (electronic supplementary material, figure S3).

**Figure 5.**
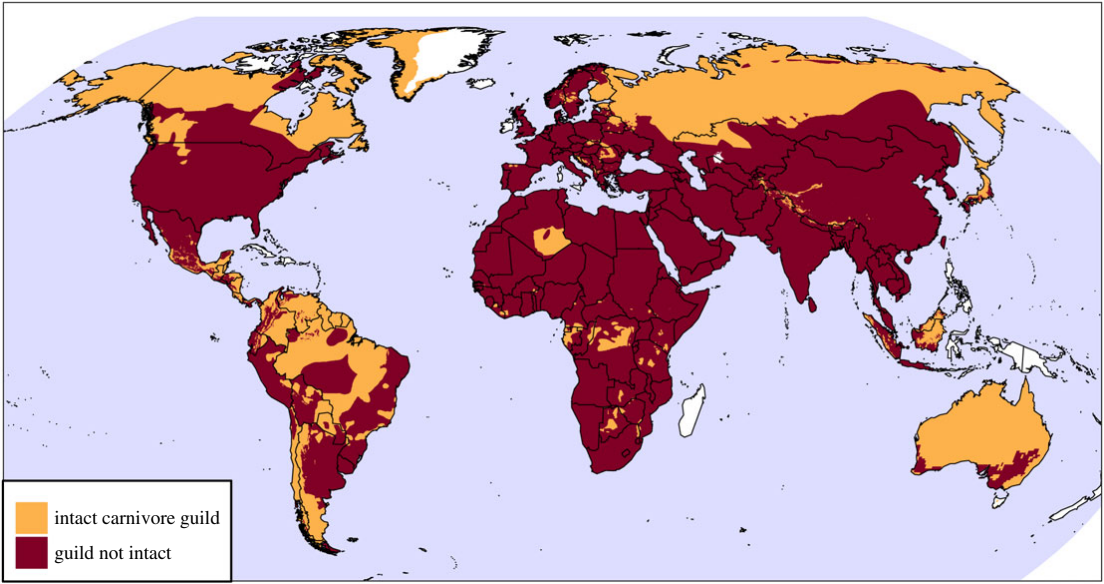
Regions of the world with intact or no longer intact large carnivore guilds (one or more species). Note that regions with high historic large carnivore richness (like Southeast Asia) seldom have intact guilds. Altogether, intact guilds make up 34% of the world's land area while 96% of land (excluding Antarctica) once contained one or more large carnivores.

Globally, large carnivores historically covered 96% of the world's land area, but intact large carnivore guilds now occupy just 34% of the world's land (figure 5). The proportion of land covered by intact guilds varied substantially by region: Oceania (89%), Europe including Russia (57%), Americas (48%), Southeast Asia (37%), Africa (8%), Asia excluding Southeast Asia (5%).

Our model results indicate that rural population density (average: 19 people per km^2^, s.d.: 48 people per km^2^), cattle density (average: 9.9 cattle per km^2^, s.d.: 26 cattle per km^2^) and cropland (average: 12%, s.d: 19%) were all positively associated with large carnivore range contractions (*p* < 0.0001) (electronic supplementary material, figure S4). The estimated increases (with 95% confidence intervals) in the odds of large carnivore range contraction for 1 s.d. increase in (log 1 plus transformed) rural population density and cattle density were 20% (17%, 24%) and 24% (21%, 27%), respectively. The estimated increase in the odds of range contraction per one standard deviation increase in percentage cropland was 72% (68%, 75%). The random intercepts by species explained 46.0% of the variability in the response (marginal *R*^2^: 29.1%, conditional *R*^2^: 75.2%). The inclusion of random intercepts and slopes by geographical region (regions listed in figure 4) increased the conditional pseudo-*R*^2^ by 3.95% (from 75.18% to 79.13%). The random effect conditional modes (electronic supplementary material, figure S5) indicate substantial variation by geographical region in the effect sizes.

## 4. Discussion

### 4.1. Individual maps

While range contractions of well-studied large carnivores like tigers are often noted in the literature [29], our results show that major range contractions are common for most of the 25 large carnivores (figure 1). Eighty eight per cent (22/25) of the large carnivores had range contractions of 20% or more (figure 2). This is substantially more than the reported 40% (17/43) of North American carnivores and ungulates with range contractions of at least 20% [9]—a difference probably due to the greater vulnerability of large carnivores to anthropogenic threats, many of which continue today.

By giving historical context to current ranges, our results show the extent to which large carnivores have been extirpated from Europe despite recent reports of large carnivore recoveries there [30]. In certain cases, range contraction patterns appear to have occurred due to unusual, species-specific circumstances. For example, the red wolf's current range is due to a planned re-introduction and the dingo's current range is limited by barrier fencing spanning across parts of Australia. Moreover, the dingo's historic range is itself due to an introduction several thousand years ago. The unique circumstances for each large carnivore mean care must be taken when trying to interpret range contractions at the level of individual species. Therefore, we have emphasized composite range contraction maps and figures showing broad trends, changes in guilds containing many species, and modelling range contractions for all large carnivores pooled together.

### 4.2. Family and body mass

The finding that species' taxonomic family and body mass do not appear to be strongly predictive of range contraction extent (electronic supplementary material, figure S2) appears to contradict previous work showing that extinction risk is higher for larger-bodied carnivore species [3,31]. It is possible that the lack of apparent relationship between species body mass and percentage range contraction is due to the limited number of observations (only 25 large carnivores), which makes statistical hypothesis testing at the level of individual species difficult, particularly once spatial and phylogenetic dependence have been modelled. On the other hand, the lack of relationship could mean that extrinsic environmental factors (e.g. human, cattle density) are more predictive of range contractions than intrinsic factors (e.g. body mass). This is consistent with the more limited body mass range spanned by large carnivore species relative to the ranges spanned by the taxa considered in previous analyses [3,31].

### 4.3. Composite maps and results

As expected, historic and current large carnivore richness are strongly correlated spatially (figure 3). Large regions of the eastern United States and Europe have lost 100% of their large carnivores (figure 3). The fact that this level of loss has not occurred in many other regions with high HPD suggests that there are other key drivers of carnivore conservation outcomes (e.g. human culture and intensive livestock), as supported by our model results (electronic supplementary material, figure S4). The biomes where the lowest loss in mean large carnivore richness occurred tend to be those with low rural population densities and limited agriculture, consistent with general human impacts (including agriculture) being linked to range contractions (electronic supplementary material, figures S1 and S3) [32].

### 4.4. Guild analysis

Large carnivore guilds, particularly those outside the far North have undergone substantial reductions in area since historical times (figure 5). This is notable as conservation may be more readily accomplished at the level of whole predator guilds. While carnivore species abundances may vary inversely due to competitive exclusion [33], there are also well-documented facilitative relationships among large carnivore species. For example, through trophic cascades, grey wolves can indirectly benefit berry-producing shrubs, providing food for grizzly bears [34]. Many large carnivores (e.g. brown hyenas) benefit from scavenging carcasses left by other carnivore species [35]. Another intriguing possibility is that of interspecific cooperation among large carnivores. Grey wolves and striped hyenas have been documented travelling together, possibly cooperating to benefit from the wolves' superior ability to subdue large prey and the striped hyenas' better sense of smell and ability to break large bones [36]. Facilitative interactions among large carnivores mean that the extirpation of one or more species could negatively impact the others. Another reason to attempt conservation at the scale of guilds is that it is often easier to focus conservation efforts around certain ‘flagship’ large carnivore species. While some of the most popular flagship species are large carnivores (e.g. grey wolves and tigers), not all large carnivores are well recognized [37]. Conservation programmes centred around flagship large carnivores that maintain adequate habitat, reduce trapping and protect shared prey base may benefit some of the lesser known large carnivores like clouded leopards [2].

When assessing the coverage of intact carnivore guilds, we treated co-occurring large carnivores as forming a single guild. However, these species can also be divided into functional groups such as ‘bone crushers’, ‘stalk and ambush carnivores’, and ‘pursuit carnivores’ based on their method of hunting and other characteristics [38]. Our treatment of co-occurring large carnivores as forming a single guild allows for the possibility of complex emergent predator effects that span multiple functional groups [38]. For example, co-occurring wolves and brown bears have well-documented emergent effects despite being in different functional groups [39].

### 4.5. Model results

The strong positive estimated effect of cattle density on the likelihood of range contraction (electronic supplementary material, figure S4) is consistent with the literature on large carnivore conservation and livestock. There are several mechanisms by which cattle and other extensively grazed livestock can adversely impact large carnivores. Cattle compete with wild ungulates, potentially reducing the availability of the carnivores' natural prey. This prey depletion leads to less food available for carnivores, reducing their abundances and possibly leading to increased human–carnivore conflict related to livestock depredation [2]. Similarly, loss of prey base was also probably a major driver of the Pleistocene large carnivore range contractions and extinctions as megaherbivores in many regions appear to have been primarily predator-limited prior to the arrival of human hunters [40]. Regardless of prey availability issues, real or perceived risks to livestock may lead humans to persecute carnivores [41]. The conversion of natural landscapes to cropland reduces the availability of wild prey for carnivores and brings them into closer contact with humans, helping to explain the observed positive association between cropland and range contractions. Higher rural population density may also put humans and carnivores in close contact, consistent with the positive estimated effect [10].

Human tolerance of large carnivores, government policy and other social factors are probably very important predictors of range contractions, but we lacked the data to assess their effects. Even in areas with substantial livestock and cropland, carnivores may be able to persist depending on human attitudes. For example, leopards and spotted hyenas were found to persist in a cropland-dominated region of western Maharashtra, India with more than 300 people per km^2^ [42]. Similarly, spotted hyenas have been observed in highly populated regions of Ethiopia despite a lack of natural prey as they are able to subsist on garbage and livestock [43].

### 4.6. Limitations

There are several key limitations associated with our use of historic range maps. These maps tend to have low resolution, not showing holes in species' historic ranges or small ‘islands’ [9]. We attempted to deal with this limitation by focusing on broad patterns and trends in species' range contractions, potentially mitigating issues associated with fine-scale range map accuracy. In addition to their coarse scale, the range maps do not include information on species abundances, which can vary greatly across species' ranges. This means that we were unable to assess changes in regions with ecologically effective predator densities—a key benchmark for conservation success [44]. There may also be variation in both historic and current range map accuracy from species to species, with particularly coarse historic range maps being associated with overestimates of percentage range contraction. The extents of range contractions may also be overestimated by the lack of range expansions in our core map set (figure 1). Although large carnivore ranges may have expanded in some cases, we found no evidence of substantial range expansions (relative to the year 1500) in the literature. Most of the apparent range expansions in the raw range map set were very small and likely to be the result of mapping errors, making potential range expansions a relatively minor source of error in our analysis. Extending the historic ranges to cover areas of apparent expansion resulted in a median increase in raw historic range area of 0.13% (maximum increase: 3.3%). Our range map set is also limited in that it does not show regions of hybridization (arguably a form of range contraction), which are important for canid species such as dingoes [45] and red wolves [46].

The modelling portion of our analysis has an additional limitation in that model covariates (human and cattle density and cropland) are relatively current, while the range contraction process may have begun centuries ago. However, our results may still be interpretable, as carnivore ranges have probably contracted the most within the last hundred years and current covariate values are probably strongly correlated with past values. That is, regions with high rural population density today probably had relatively high rural population density in the recent past and so on. We have focused on models at the global scale (using all ranges together) to avoid the possibility of spurious correlations that could occur when fitting models at the level of individual species due to inaccuracies in individual range maps. The model (and other results) apply only to the species in our analysis and thus may not be relevant to semi-aquatic large carnivores, medium-sized carnivores or other taxa.

### 4.7. Conservation implications

This analysis provides several key insights into how best to conserve threatened large carnivore populations. The general lack of relationship between life-history traits and range contraction means that most large carnivore species are potentially at risk of range contraction and other associated drivers of extinction risk (e.g. population declines). As many carnivores were historically sympatric and are at high risk of future range contraction, conservation should be accomplished at the level of whole predator guilds when possible. Conservation of entire predator guilds has the added benefit of maintaining important species interactions and emergent ecological effects caused by co-occurring predators. Guild conservation can be accomplished, for example, by expanding and strengthening protected area networks or by increasing human tolerance of predators. Although increasing rural human population densities are linked to range contractions and significant future population increases are projected, many large carnivores are resilient, particularly when human attitudes and policy favour their conservation. This helps to explain the large carnivore recoveries observed in Europe and elsewhere (e.g. grey wolves in the continental United States). Similarly, although our results associate increasing cropland and cattle density with range contractions, this relationship may be limited when predator-friendly agriculture methods are employed—an area where more research and practice is needed. Ultimately, changes in species' ranges are ongoing, dynamic processes and, in the face of newer threats like anthropogenic climate change, it is critical to continue to monitor large carnivore ranges to ensure the future of these species. Our analysis serves as a starting point for this by providing an accurate measure of the historic and current status of the world's largest carnivores.

## Supplementary Material

Large Carnivore Range Contractions - Supplementary tables and figures

## Data accessibility

Historic range map sources and range contraction statistics are given in electronic supplementary material, tables S1 and S2.
