# Supplementary material for: Range contractions of the world's large carnivores
Source: R Soc Open Sci. 2017 Jul 12;4(7):170052. doi: 10.1098/rsos.170052 (PMC5541531; doi:10.1098/rsos.170052)
Supplement: Large Carnivore Range Contractions - Supplementary tables and figures [file rsos170052supp1.docx]

**Supporting Information**

**Table S1.** Sources of the historic range maps used in our analysis. Elevation limits (lower and upper) are in meters. Modifications to the source maps are listed in the footnotes.

| **Scientific Name** | **Common Name** | **Map source** | **Lower** | **Upper** |
| --- | --- | --- | --- | --- |
| *Acinonyx jubatus* | Cheetah | Morrison et al. (2007) |  | 4000 |
| *Canis lupus* | Gray wolf | Morrison et al. (2007)^[[1]](#footnote-1)^ |  | 2400 |
| *Canis lupus dingo* | Dingo | Letnic et al. (2012) |  |  |
| *Canis rufus* | Red wolf | Morrison et al. (2007) |  | 2400 |
| *Canis simensis* | Ethiopian wolf | Ray et al. (2005) |  |  |
| *Crocuta crocuta* | Spotted hyena | Ray et al. (2005) |  | 4100 |
| *Cuon alpinus* | Dhole | IUCN canid action plan^[[2]](#footnote-2)^ |  | 5300 |
| *Helarctos malayanus* | Sun bear | Morrison et al. (2007) |  | 2100 |
| *Parahyaena brunnea* | Brown hyena | Ray et al. (2005) |  | 1500^[[3]](#footnote-3)^ |
| *Hyaena hyaena* | Striped hyena | Morrison et al. (2007) |  | 3300 |
| *Lycaon pictus* | African wild dog | Ray et al. (2005) |  | 4000 |
| *Lynx lynx* | Eurasian lynx | IUCN felid action plan^[[4]](#footnote-4)^ |  | 5500 |
| *Melursus ursinus* | Sloth bear | Morrison et al. (2007) |  | 2000 |
| *Neofelis diardi* | Sunda clouded leopard | https://commons.wikimedia.org/wiki/  File:Clouded_leopard_prevalence.png | | 1500 |
| *Neofelis nebulosa* | Clouded leopard | https://commons.wikimedia.org/wiki/File:  Clouded_leopard_historic_prevalence.png | | 3000 |
| *Panthera leo* | Lion | Morrison et al. (2007) |  | 4200 |
| *Panthera onca* | Jaguar | Panthera |  | 3000 |
| *Panthera pardus* | Leopard | Morrison et al. (2007) |  | 5200 |
| *Panthera tigris* | Tiger | Panthera |  | 4500 |
| *Panthera uncia* | Snow leopard | Morrison et al. (2007) | 750 | 5800 |
| *Puma concolor* | Puma | Panthera |  | 5800 |
| *Tremarctos ornatus* | Andean black bear | Morrison et al. (2007) | 250 | 4750 |
| *Ursus americanus* | American black bear | Morrison et al. (2007) ^[[5]](#footnote-5)^ |  | 3500 |
| *Ursus arctos* | Brown bear | Morrison et al. (2007) |  | 5000 |
| *Ursus thibetanus* | Asiatic black bear | Morrison et al. (2007) |  | 4300 |

**Table S2.** Summary data for large carnivore range contractions. Historic, current, and lost range areas are in square kilometers. “% Lost” is the estimated percentage range contraction.

| **Scientific Name** | **Common Name** | **Historic** | **Current** | **Lost** | **% Lost** |
| --- | --- | --- | --- | --- | --- |
| *Canis rufus* | Red wolf | 2,205,755 | 5,771 | 2,199,984 | 99.7% |
| *Canis simensis* | Ethiopian wolf | 888,157 | 6,187 | 881,969 | 99.3% |
| *Panthera tigris* | Tiger | 16,622,223 | 780,646 | 15,841,576 | 95.3% |
| *Panthera leo* | Lion | 26,914,341 | 1,703,935 | 25,210,406 | 93.7% |
| *Lycaon pictus* | African wild dog | 18,924,058 | 1,278,603 | 17,645,455 | 93.2% |
| *Acinonyx jubatus* | Cheetah | 34,997,842 | 2,964,276 | 32,033,567 | 91.5% |
| *Cuon alpinus* | Dhole | 19,321,239 | 3,396,114 | 15,925,125 | 82.4% |
| *Panthera pardus* | Leopard | 41,990,824 | 8,647,609 | 33,343,215 | 79.4% |
| *Panthera uncia* | Snow leopard | 5,440,630 | 1,216,226 | 4,224,404 | 77.6% |
| *Tremarctos ornatus* | Andean black bear | 1,190,733 | 294,980 | 895,753 | 75.2% |
| *Neofelis nebulosa* | Clouded leopard | 4,950,609 | 1,798,205 | 3,152,404 | 63.7% |
| *Ursus thibetanus* | Asiatic black bear | 8,645,920 | 3,142,301 | 5,503,619 | 63.7% |
| *Neofelis diardi* | Sunda clouded leopard | 1,184,443 | 582,239 | 602,204 | 50.8% |
| *Helarctos malayanus* | Sun bear | 3,285,879 | 1,628,906 | 1,656,973 | 50.4% |
| *Panthera onca* | Jaguar | 17,774,713 | 8,818,148 | 8,956,565 | 50.4% |
| *Ursus arctos* | Brown bear | 46,588,688 | 26,891,864 | 19,696,824 | 42.3% |
| *Melursus ursinus* | Sloth bear | 2,481,079 | 1,518,776 | 962,304 | 38.8% |
| *Ursus americanus* | American black bear | 15,829,679 | 9,714,012 | 6,115,666 | 38.6% |
| *Puma concolor* | Puma | 31,259,847 | 21,240,084 | 10,019,763 | 32.1% |
| *Parahyaena brunnea* | Brown hyena | 2,876,377 | 2,093,842 | 782,535 | 27.2% |
| *Canis lupus* | Gray wolf | 64,169,543 | 47,702,213 | 16,467,330 | 25.7% |
| *Crocuta crocuta* | Spotted hyena | 19,146,204 | 14,541,846 | 4,604,359 | 24.0% |
| *Hyaena hyaena* | Striped hyena | 27,675,695 | 23,510,432 | 4,165,263 | 15.1% |
| *Canis lupus dingo* | Dingo | 7,641,425 | 6,713,643 | 927,781 | 12.1% |
| *Lynx lynx* | Eurasian lynx | 23,163,633 | 20,424,410 | 2,739,223 | 11.8% |


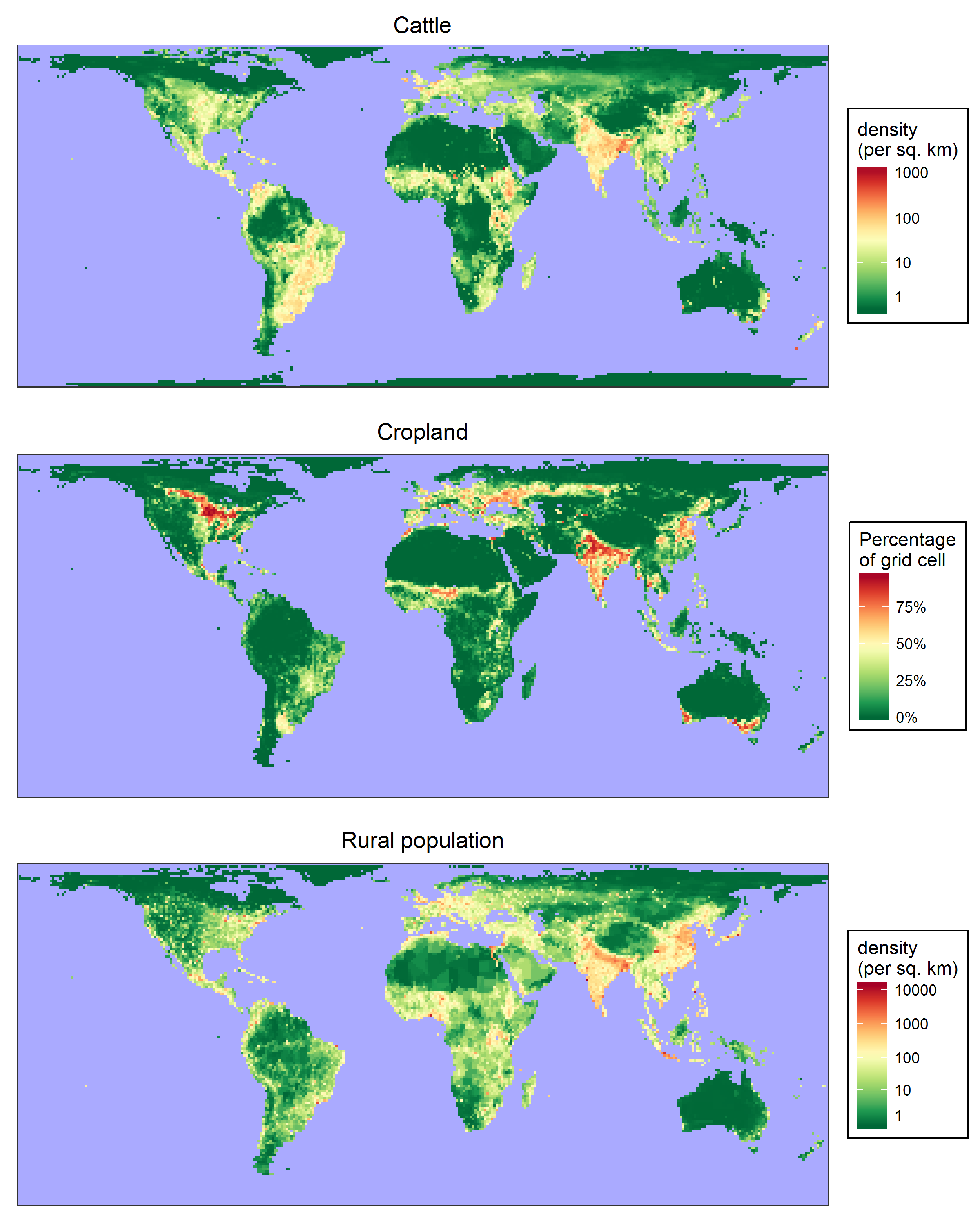


**Figure S1.** Predictor variables used in the model for predicting range contractions. For the rural population and cattle density variables, the values were log transformed (after adding 1). All variables were then scaled to have average 0 and standard deviation 1 so that their effects on the likelihood of range contraction can be compared on the same scale.


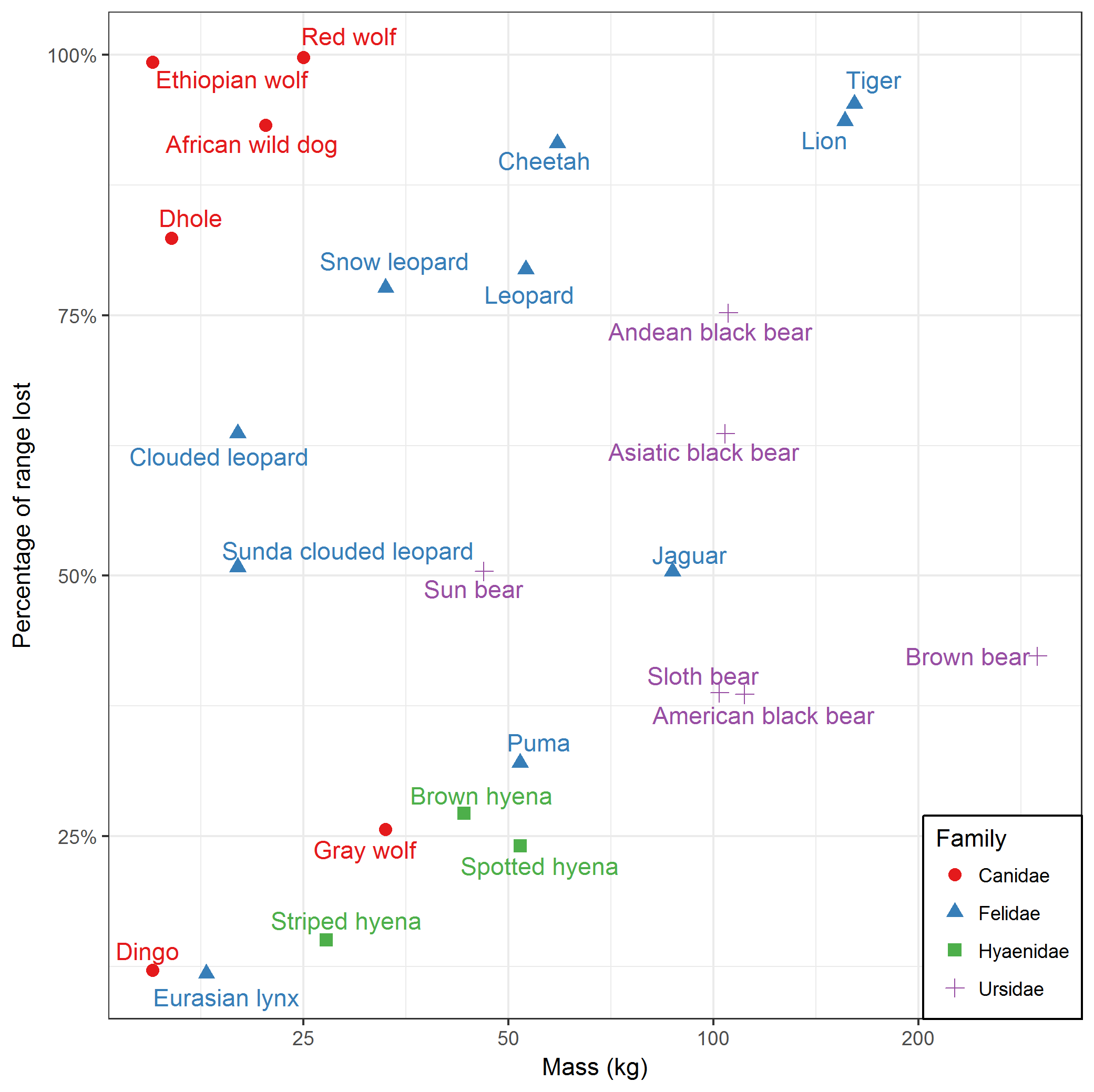


**Figure S2.** Percentage range contraction versus carnivore body mass. Overall, percentage range contraction does not appear to be closely linked to either carnivore species mass or taxonomic family.


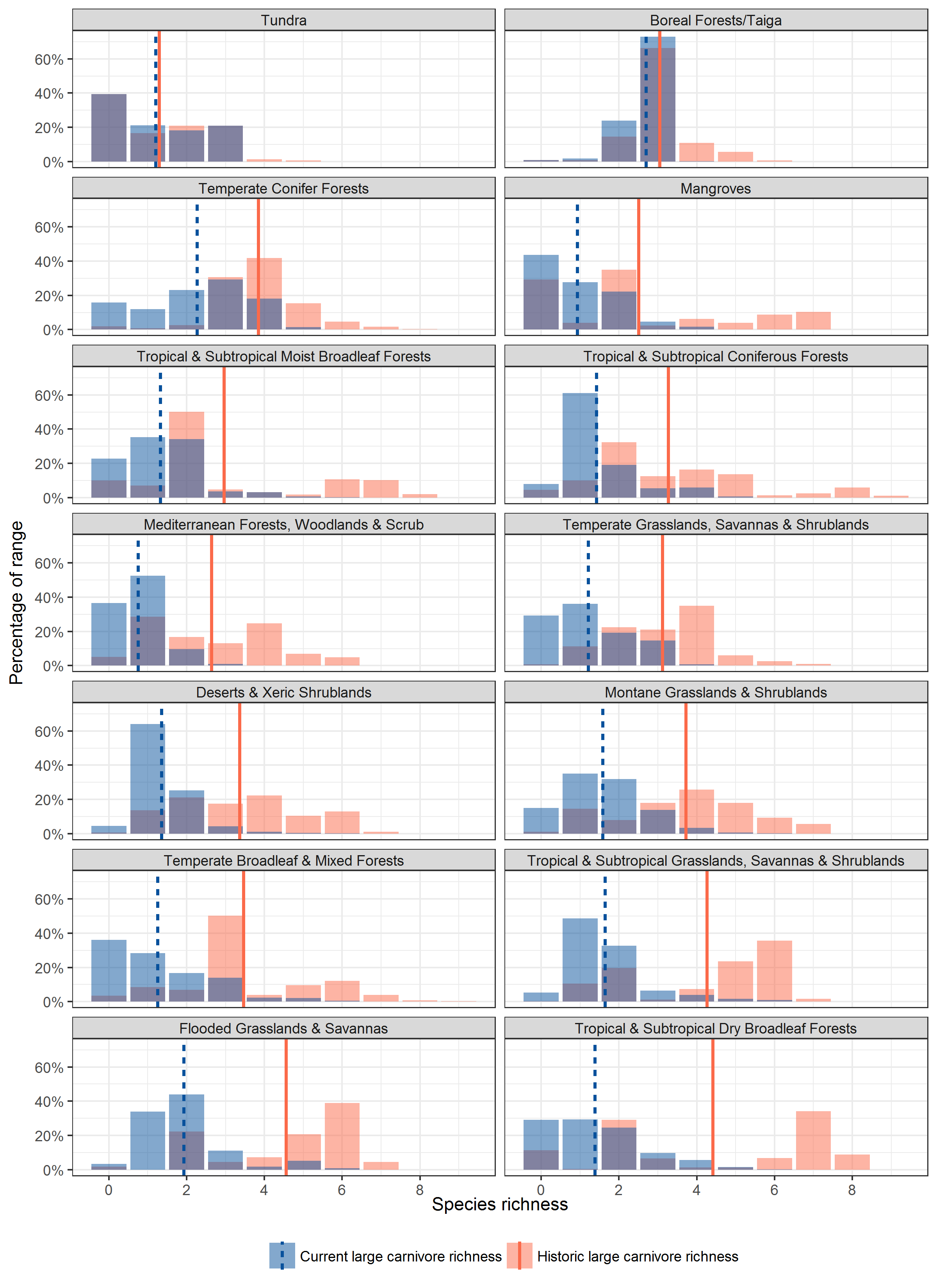


**Figure S3.** Current and historic species richness histograms by biome. Vertical lines indicate mean richness. Panels are sorted by difference in mean richness and indicate that the most extensive range contractions occurred in “Flooded Grasslands & Savannas” and in “Topical & Subtropical Dry Broadleaf Forests.” Overlap between current and historic range histogram bars is shown in dark purple.


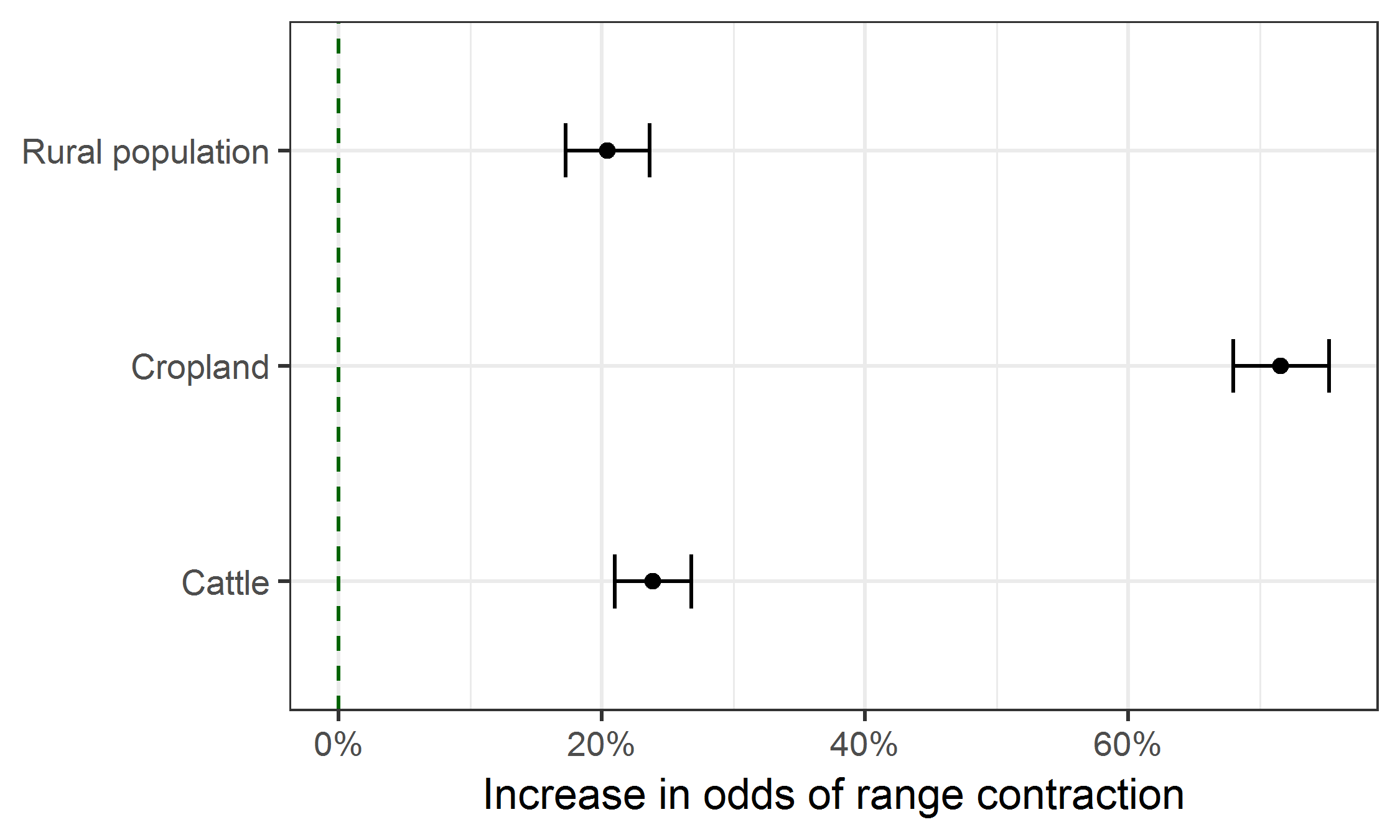


**Figure S4.** Results for a generalized linear mixed model predicting the increase in the odds of range contraction per one standard deviation increase in each predictor variable while accounting for the other variables (estimates shown with 95% confidence intervals). The p-values for all coefficients were significant (< 0.0001). To account for potential dependence the model includes random intercepts by species along with a spatial autocovariate constructed using the residuals of the corresponding non-spatial model.


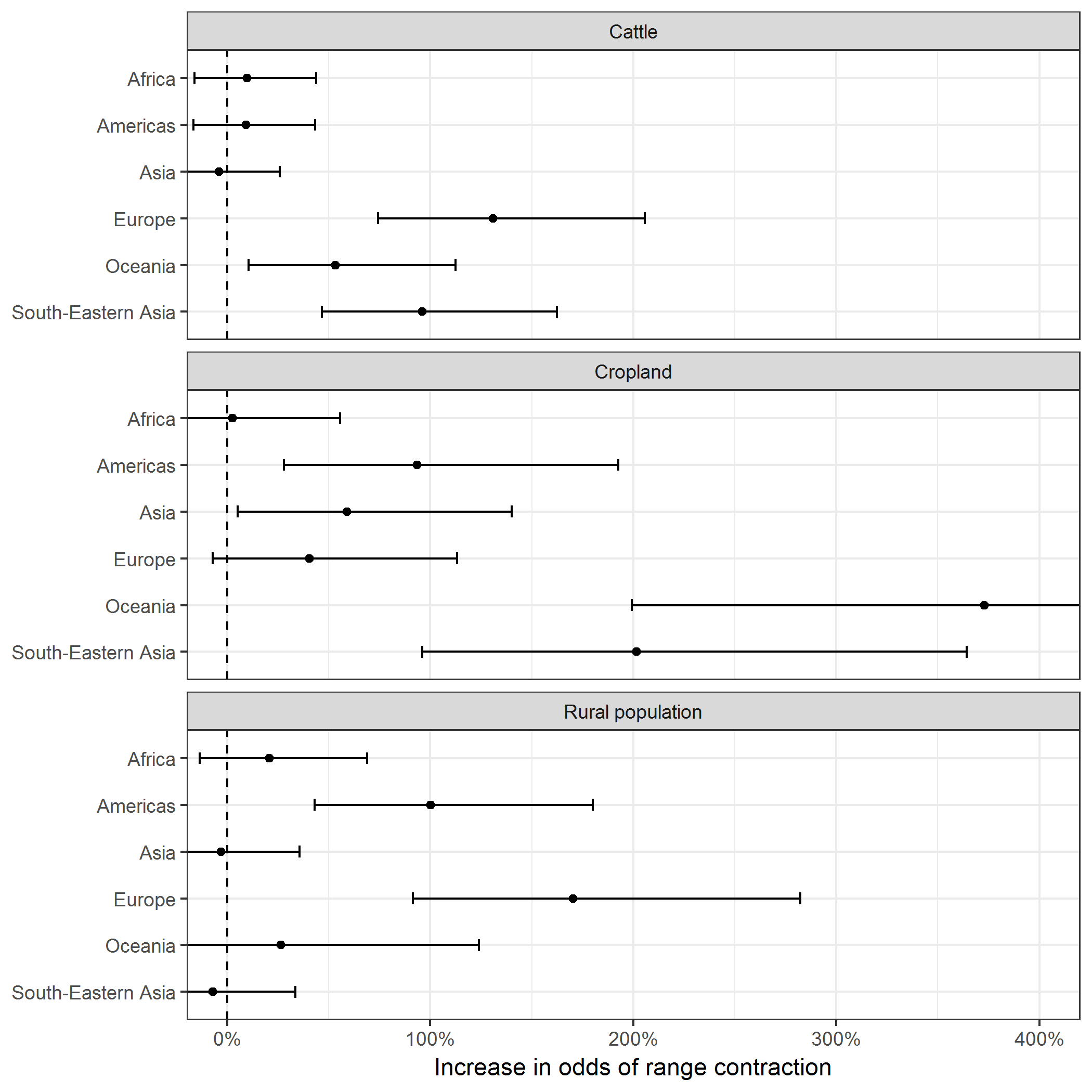


**Figure S5.** Effects of cattle density, cropland, and rural population on the odds of species range contraction by region of the world based on a spatially explicit generalized linear mixed model with random slopes (and intercepts) by geographic region and random intercepts by species. The panels show the estimated increases in the odds of range contraction per one standard deviation increase in each predictor variable (with 95% prediction intervals). All predictor variables were included together in the model. These results show substantial variation by geographic region in terms of the estimated effect sizes.

1. Added Japan, Sakhalin island (above Japan), and the corner of northeastern Russia [↑](#footnote-ref-1)
2. Extended a bit northward on the basis of the current IUCN Red List fact sheet and input from Jan Kamler [↑](#footnote-ref-2)
3. Elevation limit from the Animal Diversity Web:

   Myers P, Espinosa R, Parr CS, Jones T, Hammond GS, Dewey TA. The Animal Diversity Web (online) [Internet]. 2015 [cited 2014 Feb 27]. Available from: http://animaldiversity.ummz.umich.edu [↑](#footnote-ref-3)
4. Only for Europe (treated historic range in Asia as matching the current range, reviewed by Bodil Elmhagen) [↑](#footnote-ref-4)
5. Clipped northern portion of historic range to match with current range [↑](#footnote-ref-5)
